# BETTER LIFE- guidelines for chronic disease preventive care for people aged 18–39 years: a literature review

**DOI:** 10.1186/s12875-024-02471-9

**Published:** 2024-06-22

**Authors:** Nasheed Moqueet, Sylvie D. Cornacchi, Jesmin Antony, Ielaf Khalil, Donna Manca, Carolina Fernandes, Lawrence Paszat, Kris Aubrey-Bassler, Eva Grunfeld, Nicolette Sopcak, Andrew Pinto, Jill Konkin, Candace Nykiforuk, Linda Rabeneck, Peter Selby, Becky Wall, Mary Ann O’Brien, Aisha Lofters

**Affiliations:** 1 Public Health Agency of Canada, Ottawa, ON Canada; 2 McMaster University, Hamilton, ON Canada; 3 Women’s College Hospital, 76 Grenville St, Toronto, ON M5S 1B2 Canada; 4 Lunenfeld-Tanenbaum Research Institute, Sinai Health System, Toronto, ON Canada; 5 Department of Family Medicine, University of Alberta, Edmonton, AB Canada; 6 Sunnybrook Research Institute, Sunnybrook Health Sciences Centre, Toronto, ON Canada; 7 Primary Healthcare Research Unit, Memorial University of Newfoundland, St. John’s, NL Canada; 8 Ontario Institute for Cancer Research, Toronto, ON Canada; 9 Li Ka Shing Knowledge Institute, St. Michael’s Hospital, Toronto, ON Canada; 10 School of Public Health, University of Alberta, Edmonton, AB Canada; 11 Institute of Health Policy, Management and Evaluation, University of Toronto, Toronto, ON Canada; 12 Centre for Addiction and Mental Health, Toronto, ON Canada; 13 Department of Family and Community Medicine, University of Toronto, Toronto, ON Canada; 14 Durham Region Health Department, Whitby, ON Canada

**Keywords:** Prevention, Screening, Health promotion

## Abstract

**Background:**

The original ‘BETTER’ (Building on Existing Tools To Improve Chronic Disease Prevention and Screening in Primary Care) approach consisted of a prevention-focused visit between participants aged 40–65 years and a “Prevention Practitioner” (PP), who empowered the participant to set achievable prevention and screening goals for cancers and chronic diseases. BETTER was successfully adapted for economically deprived communities (BETTER HEALTH) in Canada. Our objective was to conduct a review of guidelines in preparation for adapting the ‘BETTER HEALTH’ approach for younger adults aged 18–39 years living with lower income, a group known to have earlier mortality due to a higher prevalence of preventable chronic diseases than their peers with higher income.

**Methods:**

We searched multiple electronic databases and grey literature for clinical practice guidelines on prevention/screening and included those that met the following criteria: published in English from 2008–2020 in Canada or any of the following countries (Australia, Ireland, New Zealand, Scotland, United States and England); and addressed prevention or screening. We assessed quality using the Appraisal of Guidelines for Research and Evaluation (AGREE) II tool and extracted data (publication details, recommendations, and Quality/Level of evidence as reported by authors) from sources with overall scores of 5 or higher. Final recommendations were compiled after harmonization with input from diverse stakeholders (co-investigators, PPs, and the Community Advisory Committee).

**Results:**

We included a total of 85 guidelines, and developed a final list of 42 recommendations for 18–39 year-olds across 21 topics. Specific recommendations fell under the following topics: *cancers, cardiovascular disease, diabetes, obesity, lifestyle (alcohol; healthy nutrition/physical activity); healthy relationships and healthy sexuality, immunization, oral health, social determinants of health, and substance use*.

**Conclusion:**

We identified evidence-based guidelines on individual-level prevention/screening actions for adults 18–39 years old and relevant for those living with lower income which will directly inform development and implementation of the BETTER LIFE intervention.

**Supplementary Information:**

The online version contains supplementary material available at 10.1186/s12875-024-02471-9.

## Introduction

Despite the existence of strong evidence for lifestyle modifications and for screening and preventive actions to improve health outcomes, an implementation gap exists due to limited physician time [1], conflicting/unclear guidelines, and difficulties inherent to sustained behaviour change [2]. The original BETTER (Building on Existing Tools To Improve Chronic Disease Prevention and Screening in Primary Care) intervention was designed to address this gap by providing an integrated approach to increasing uptake of chronic disease prevention and screening (CDPS) actions using a framework of shared decision-making between patient and practitioner. In a pragmatic cluster randomised control trial (RCT), the BETTER approach improved the uptake of CDPS actions against heart disease, diabetes and several cancers (colorectal, breast and cervical cancers) by 32.5% in urban primary care settings in Alberta and Ontario, Canada [2, 3]. The intervention consisted of an individual prevention-focussed visit between participants aged 40–65 years and a “Prevention Practitioner” (PP), who used principles of motivational interviewing to empower the participant to set achievable prevention and screening goals, based on the harmonization of evidence, which were then recorded in a goals sheet and a personalized ‘prevention prescription’.

There have been subsequent modifications of the BETTER approach with similar positive results. ‘BETTER 2’ targeted the same age group as the original BETTER but modified the approach for different populations due to equity concerns, including individuals from rural, lower income, or historically marginalized backgrounds in Newfoundland and Labrador and the Northwest Territories, Canada [4]. Subsequently, BETTER WISE (Building on Existing Tools to Improve Cancer and Chronic Disease Prevention and Screening in Primary Care for Wellness of Cancer Survivors and Patients) tailored the BETTER approach for cancer survivors (breast, colorectal, prostate) aged 40–65 and also included screening for poverty, as well as an updated literature review to recommend specific prevention and screening actions [5]. Another modified version, BETTER HEALTH: Durham used a public health-led model with public health nurses serving as PPs for 40–64 year-olds living with lower income in Durham, Ontario, and found a 53% increase in completed health actions (immediate intervention, n = 60 vs. wait-listed arm, n = 66) [6, 7]. Although there were positive results for this age group, the community advisory group for BETTER HEALTH: Durham suggested that starting the intervention at 40 years of age was too late for people living with low income, where evidence shows an earlier onset of chronic diseases [8]. We aimed to adapt the BETTER HEALTH: Durham intervention to a new population of adults aged 18–39 years living with low income, a group known to have earlier mortality due to, and higher prevalence of, preventable chronic diseases than their peers with higher income.

To support the adaptation, we conducted a review of guidelines to identify and assess prevention and screening actions for health issues and risk factors amenable to individual change for the 18–39 year age group. This paper describes the methods and results of the literature review.

## Methods

### Overview of search strategy

**Fig. 1:**
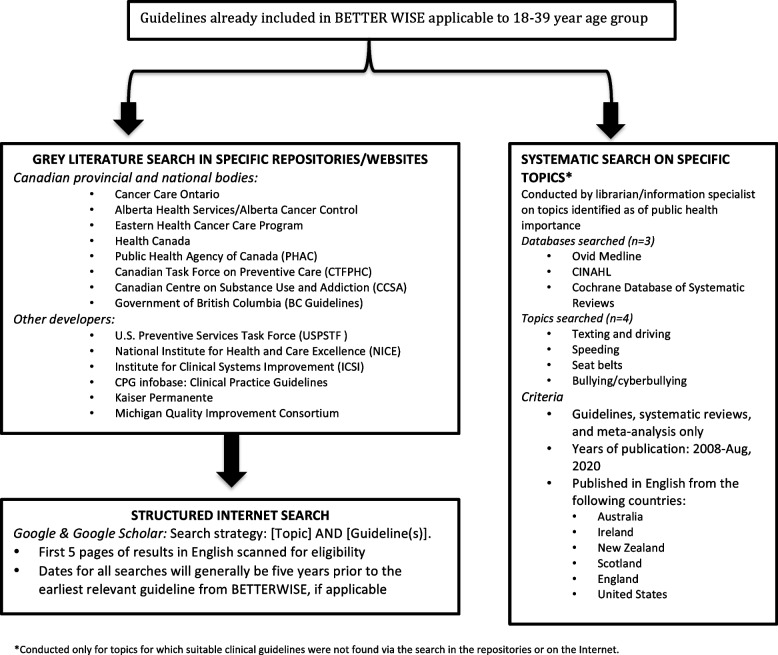
Search strategy for guidelines for BETTER LIFE

First, we assessed the data sources (clinical practice guidelines) from the most recent BETTER WISE study [9], which had entailed a rigorous evidence review process to recommend specific prevention and screening actions, for applicability to adults aged 18–39 years.Then, we used a structured grey literature search of specific repositories and websites to find relevant clinical practice guidelines for new topics suggested by the research team. If guidelines were unavailable for these topics, we performed a systematic literature search in the databases Ovid Medline, CINAHL (Cumulated Index to Nursing and Allied Health Literature), and the Cochrane Database of Systematic Reviews to identify systematic reviews/meta-analyses. Thus, our search and eligibility criteria for new sources was restricted to clinical practice guidelines (i.e. excluding systematic reviews, meta-analyses, and review of reviews when guidelines were found) and expanded to allow systematic reviews and meta-analyses when guidelines were not available (See Fig. 1).

### Search strategy for topics of interest

To create the overall search strategy, we consulted an experienced information specialist (CZ). We used different combinations of key words such as ‘guidelines’, ‘chronic disease prevention’, ‘prevention’, ‘clinical practice guidelines’, and ‘screening’ with terms from topics of interest from previous versions of BETTER (*cardiovascular disease, diabetes, cancer, obesity, diet and nutrition, physical activity, smoking/tobacco and alcohol use)* and new topics suggested by the wider research team (co-investigators, PPs, Community Advisory Committee (CAC)) due to their importance for our target population (See Supp Table 1).

### Search sources

We conducted a structured search in repositories of guidelines at the provincial level (Ontario, Alberta, Newfoundland & Labrador): Cancer Care Ontario; Cancer Control Alberta; Eastern Health Cancer Care Program; and national level: Health Canada; Public Health Agency of Canada (PHAC); and the Canadian Task Force on Preventive Health Care (CTFPHC). (Details in Fig. 1).

We did not find guidelines for four topics recommended by our study team for our target population (*speeding, texting & driving, seat belts, bullying & cyberbullying).* Therefore, we then conducted a systematic search on select databases (Ovid Medline, CINAHL, Cochrane Database of Systematic Reviews) for systematic reviews and meta-analyses published from 2008-August 2020 on these topics.

### Inclusion & exclusion criteria

When screening abstracts obtained from our searches, we included articles for full-text review if they met the following criteria: clinical practice guidelines in English only; published from 2008–2020; country of publication was Australia, Canada, England, Ireland, New Zealand, Scotland, or US; included at least one of the identified topics in title or abstract; and addressed prevention or screening.

At full-text screening, we excluded articles if they met any of the following: exclusively focused on management or treatment; exclusively targeted ages not 18–39 years old (i.e., under 18, 40 or older); lacked individual-level recommendations (i.e. contained only macro-level data (e.g. legal, policy)); or lacked evidence of synthesis. With the exception of the four topics covered during the systematic search, we also excluded full-texts if they were systematic reviews, review of reviews, or meta-analyses.

During full-text screening, if multiple eligible sources existed, we used a hierarchical approach to determine inclusion: preference for most recent Canadian guideline/ review and if not available, relevant guidelines from any of 6 aforementioned primarily English-speaking countries of interest. If there were discrepancies or disagreements among guidelines, we searched for and extracted information from primary or common references.

All abstracts and full-texts were uploaded and screened using Covidence [10].

### Quality assessment

We chose AGREE-II for quality assessment since it was developed specifically for assessing quality of *existing* practice guidelines, unlike GRADE (Grading of Recommendations, Assessment, Development, and Evaluations), which is most suited for developing guidelines de novo and for rating primary sources of evidence for specific outcomes, which was outside the scope of our study. We used a two-step process to assess guideline quality. For the first step, two trained reviewers (NM and SC) independently used a shorter 2-item AGREE-II [11] rating system to assess the “Rigour of development” (items 7 and 12—‘*Systematic methods were used to search for evidence’* and *‘There is an explicit link between the recommendations and the supporting evidence’*, respectively) on all references. If methodological details were missing from guidelines, we emailed authors or guideline developers to request more information. Both reviewers had to assign a score of 4 or higher (out of 7) on both AGREE-II items for the article to move to full quality assessment with the 23-item AGREE-II tool.

Specifically, the reviewers examined the ‘Methods’ section of each guideline to assess the details of systematic methods (Item #7) that were used and consulted any methods papers that governed the overall initiative when available [12–20]. If the guideline developer did not report any evidence of an independent synthesis as per the first step in the AGREE–II screening process, the guideline was not assessed further. If no Canadian reference met the criteria for the 2-item AGREE-II screening tool on a given topic, the reviewers then assessed the quality of the non-Canadian documents. Disagreement over scores was discussed and a final decision was determined by consensus.

For step 2, two reviewers independently applied the full AGREE-II instrument on all guidelines that passed the 2-item screening. Overall scores of 5 and above (out of 7) by both reviewers were used to move to full data extraction phase. To ensure consistent interpretation of data quality, we pilot tested the full AGREE-II tool on 5 articles that had previously been included in BETTER WISE and that also met the eligibility criteria for BETTER LIFE.

### Data extraction

Two reviewers also pilot tested the data extraction form on 5 articles and resolved differences by consensus. Each reviewer independently extracted data from half the included articles and then checked a subset from the other reviewer for consistency, resolving differences by discussion. Extracted data included publication details (issuing body/author, year and country of publication), participant characteristics (target population, age, ethnicity, socioeconomic metrics, identified risk factors, clinical context) and guideline details (individual-level recommendations, quality of supporting evidence, and whether conflict of interest was declared or not).

### Harmonization and synthesis of extracted data

**Fig. 2:**
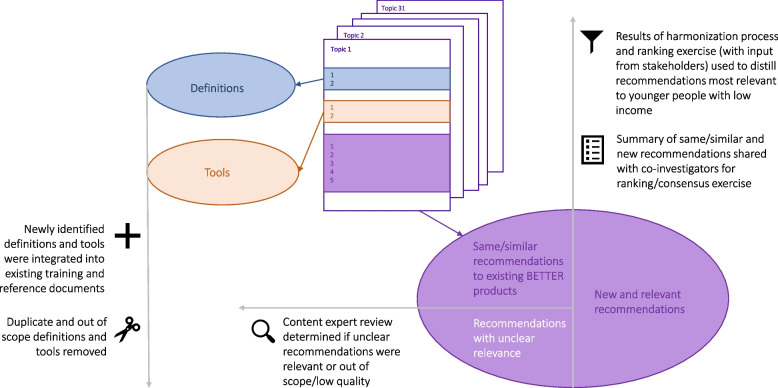
Harmonization process for BETTER LIFE

The extracted data were grouped by topics. Each article was assigned to two reviewers who independently either categorized recommendations for inclusion in BETTER LIFE or excluded them if they were duplicative, out of scope, or not actionable (See Fig. 2).

The reviewers met to discuss and assign a final primary categorization to each recommendation with the overall team meeting to resolve differences if there was no agreement between reviewers. The senior co-authors (AL and MAO) reviewed the categorizations, clarified unclear recommendations and identified specific recommendations for further review from content experts/co-investigators in the BETTER team.

### Harmonization and synthesis

We followed a similar harmonization process to Campbell-Sherer et al [9] within an overarching ADAPT-ITT framework [21].

All the co-investigators and PPs in the BETTER team were invited to provide input on topics in which they had expertise and asked to rank the newly included recommendations in an online survey (Qualtrics, Provo, UT), with the goal of reaching consensus on the top ranked (most relevant) recommendations. Recommendations ranked with a mean of 90% or above were included, while those that were that consistently ranked low (mean of less than 75%) were removed. For topics with multiple individual recommendations with mean scores of 80–89%, we combined, summarized and simplified the multiple recommendations where it seemed appropriate to do so and included them.

After the harmonization process, we compiled the final list of recommendations and topics into a table and also grouped all related included topics into existing or new ‘domains’ for data visualization.

## Results

**Fig. 3:**
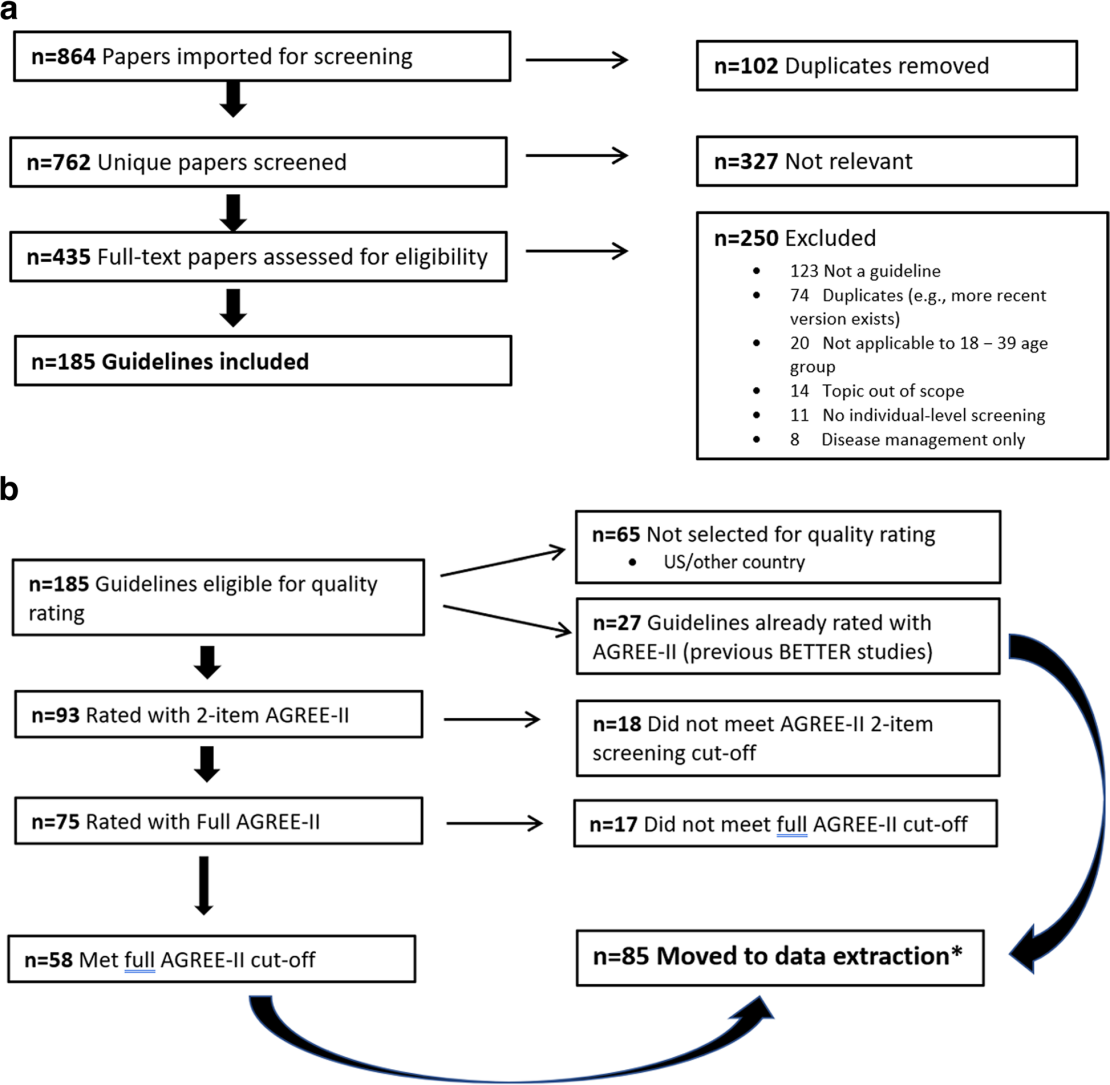
**a **Summary flow from literature search to full-text review for quality assessment. **b **Quality assessment of guidelines using the AGREE-II instrument to the data extraction stage

There were 864 abstracts, of which 762 were unique. Of these, 435 were moved to the full-text phase and assessed for inclusion. One hundred and eighty-five guidelines met the inclusion criteria for quality assessment (Fig. 3a).

From the 150 guidelines included in BETTER WISE that were published in 2008 or later, 40 guidelines were applicable to the 18–39 year age group, of which 14 had been updated since inclusion in BETTER WISE. Newer versions were available for the following 8 topics: *cancers (breast, cervical, colorectal), CVD, diabetes, obesity, lifestyle (alcohol; healthy nutrition/physical activity)*.

From the search for topics for which there were no identified guidelines *(speeding, texting and driving, seat belts, bullying and cyberbullying)*, 213 papers were uploaded into Covidence after removing duplicates. However, all the papers on these topics were excluded at various stages.

### Quality assessment

One hundred and eighty-five guidelines were eligible for quality screening (Fig. 3b). After exclusion at various stages, 93 guidelines were rated with the 2-item AGREE-II. Of these, 75 were rated with the full AGREE-II tool and 58 papers (77%, 58/75) were scored 5 or higher by both reviewers.

We extracted data from 85 guidelines (58 were new guidelines and 27 were from previous versions of BETTER). Of the 38 new topics (Supp Table 1), 22 were relevant to the 18–39 year age group (Supp Table 2).

### Harmonization and synthesis

Of the 19 colleagues invited, 9 responded, reporting expertise on atleast one of the topics on the list (between 1–8 respondents provided ranking on each of the various new recommendations). At the harmonization stage, the team removed the topic ‘*falls/injury prevention’* as the recommendation was deemed not in scope for the 18–39 age group.

Due to low ranking scores from Co-investigators, we removed 6 topics from inclusion in the final BETTER LIFE recommendations (*intimate partner violence, sexual health, skin cancer, sleep, violence, vitamins*). We also excluded *hepatitis C*as only one co-investigator provided a ranking for this recommendation, and the recommendation was to not screen for hepatitis C. On the advice of the research team, we also included screening for Adverse Childhood Experiences (ACE) [22, 23].

Based on the results of the data extraction and harmonization, the final list of topics contained 42 recommendations for 18–39 year-olds across 21 total topics (Table 1). We grouped the final list of topics into existing or new domains (See Supp Fig. 1).

**Table 1:** List of all* topics and recommendations for BETTER LIFE participants aged 18–39 years

Topic	Recommendation
Alcohol [24–28]	• Screen for unhealthy alcohol use and provide persons engaged in risky or hazardous drinking with brief behavioral counseling interventions • Do not drink in these situations: When operating any kind of vehicle, tools or machinery; using medications or other drugs that interact with alcohol; engaging in sports or other potentially dangerous physical activities; working; making important decisions; if pregnant or planning to be pregnant; before breastfeeding; while responsible for the care or supervision of others; if suffering from serious physical illness, mental illness or alcohol dependence • At risk drinker: o WOMEN: > 1 standard drinks on any one day OR > 7 drinks/week OR 3 drinks at one time o MEN: > 2 standard drinks on any one day for men OR > 14 drinks men/week OR 4 drinks at one time • Use formal assessment tools to assess the nature and severity of alcohol misuse i.e., modified Alcohol Use Disorders Identification Test (AUDIT-C)
Anxiety [29, 30]	• Screen for anxiety in those who are not currently diagnosed with anxiety disorders o Optimal screening intervals are unknown, and clinical judgement should be used to determine frequency. When screening suggests the presence of anxiety, further evaluation is necessary to establish the diagnosis and determine appropriate treatment and follow-up • Asking patients if they are feeling nervous, anxious or on edge, or whether they have uncontrollable worry can be useful to detect anxiety in patients in whom the clinician suspects an anxiety or related disorder • Screening questions from current BETTER WISE Baseline Survey (GAD-2): During the past two weeks how much have you been bothered by the following problems? 1. Feeling nervous, anxious, or on edge 2. Not being able to stop or control worrying
Breast cancer [31–34]	• Screen women with elevated risk factors—as per current provincial guidelines • Women at increased risk of breast cancer should be 'breast aware.’
Cannabis [35]	• The most effective way to avoid any risks of cannabis use is to abstain from use • Those who decide to use need to recognize that they incur risks of a variety of acute and/or long-term adverse health and social outcomes o These risks will vary in their likelihood and severity with user characteristics, use patterns, and product qualities, and so may not be the same from user to user or use episode to another
Cervical cancer [36–38]	• If there is no abnormal cytology, no personal history of cervical cancer, no personal history of a hysterectomy with removal of the cervix AND: o Patient is not immuno-compromised, pap test every 3 years o Patient is immuno-compromised, pap test every year • Transgender men who have retained their cervix should be screened according to the guidelines
Colorectal cancer [39–45]	Screen men and women with elevated risk factors as per current provincial guidelines
Contraception [46]	If the participant is sexually active, and is interested in learning more about contraception, refer to appropriate resources
Cardiovascular disease (CVD)/Hypertension [47–50]	• Hypertension screening: Blood pressure (BP) should be measured accurately in adults, at all appropriate visits, by trained healthcare practitioners (only possible at in-person visits) o When a manual office blood pressure device (MOBP) is used, hypertension is diagnosed at ≥ 140/90 o When using automated office blood pressure readings, hypertension is diagnosed when at ≥ 135/85 in the higher BP arm • Frequency of screening and BP targets differ for those with and without diabetes • Do not use a risk assessment tool to assess CVD risk in people with an estimated glomerular filtration rate (eGFR) less than 60 ml/min/1.73 m^2^ and/or albuminuria. These people are at increased risk of CVD
Depression (including suicide prevention) [51–54]	• Routinely screen all adults for depression using a standardized instrument • Always ask people with depression directly about suicidal ideation and intent. If there is a risk of self-harm or suicide: o assess whether the person has adequate social support and is aware of sources of help o arrange help appropriate to the level of risk o advise the person to seek further help if the situation deteriorates
Diabetes (including gestational diabetes mellitus) [55–59]	• All individuals should be evaluated annually for type 2 diabetes risk on the basis of demographic and clinical criteria • Repeat testing (blood glucose testing) every 3 years for women who had a pregnancy affected by gestational diabetes mellitus and normal postpartum screening test results
Folic acid [60, 61]	Women who are planning or capable of pregnancy should take a daily multivitamin supplement containing 0.4 to 0.8 mg of folic acid to prevent neural tube defects
Healthy lifestyle including nutrition [40, 41, 47, 50, 62–66] and physical activity [40, 41, 47, 64–71]	• All individuals should be encouraged to moderate energy (caloric) intake to achieve and maintain a healthy body weight and adopt a healthy dietary pattern to lower their cardiovascular disease risk o Nutritious foods are the foundation for healthy eating • Recommend at least 150 min of moderate exercise (moderate intensity includes brisk walking) or more than 75 min of vigorous physical activity per week o For additional benefits in healthy adults, a gradual increase in aerobic physical activity to 300 min a week of moderate intensity, or 150 min a week of vigorous intensity aerobic physical activity, or an equivalent combination thereof is recommended o Exercise in high-risk individuals results in CVD and mortality reductions similar to or better than reductions seen in trials for most pharmaceutical treatments • Multiple sessions of physical activity should be considered, each lasting ≥ 10 min and evenly spread throughout the week with at least two sessions including muscle strengthening activities using major muscle groups (legs, hips, back, abdomen, chest, shoulders and arms)
Immunization (includes Hepatitis B, HPV) [72–74]	• Participants should be asked if they have up to date immunization records or know the provider who would have their records
Obesity [75–80]	• Measure height, weight and calculate BMI at appropriate primary care visits, if visit is in person and participant is interested • Waist circumference screening: All patients with BMI 25 to 29.9 that have not had a waist circumference measurement in the past 2 years should have waist circumference measured • We recommended that practitioners not offer formal, structured interventions aimed at preventing weight gain in normal-weight adults. Adults who are overweight or obese may be candidates for weight-loss treatment • For adults who are obese (BMI 30–39.9) and are at high risk of diabetes, we recommend that practitioners offer or refer to structured behavioural interventions aimed at weight loss • For adults who are overweight or obese, we recommend that practitioners offer or refer to structured behavioural interventions aimed at weight loss • Patients with certain risk factors (family history of diabetes, personal history of gestational diabetes or polycystic ovarian syndrome, or being a member of certain racial/ethnic groups (African American, American Indian or Alaskan Native, Asian American, Hispanic or Latino, or Native Hawaiian or Pacific Islander)) may also be at increased risk of diabetes at a younger age (age < 40) or at a lower BMI and should be considered for Diabetes screening
Oral Health [81]	• Brush at least twice daily, with a fluoridated toothpaste • Brush last thing at night and at least on one other occasion • Use fluoridated toothpaste (1350 – 1500 ppm fluoride) • Spit out after brushing and do not rinse, to maintain fluoride concentration levels
Parenting [22, 82]	• Parents should be discouraged from using corporal or physical punishment because of its negative impact on a child’s behavior and mental health
Social determinants of health [22, 83–85]	• Ask screening questions about housing and food insecurity, adverse childhood experiences, and social supports
Sexually transmitted infections (STI) [86–89]	Conduct a brief risk assessment on all individuals to quickly identify or rule out major risk factors associated with increased risk of STIs
Smoking/Tobacco [47, 64, 65, 68–70, 90–92]	• Ask all adults about tobacco use • Advise them to stop using tobacco • Avoid passive smoking • Provide referral to behavioral interventions or PCP for approved pharmacotherapy for cessation to adults who use tobacco
Substance use [93, 94]	Screen all participants to determine whether they use substances
Vaping [95, 96]	• Screen everyone for vaping/ENDS (electronic nicotine delivery systems) use • Those who smoke or vape should be advised to quit (or cut down) tobacco and ENDS use and be referred if interested for evidence-based options for control of nicotine addiction, including counselling and pharmacologic strategies

*The 21 topics in this table include both new topics and those from previous versions of BETTER. Tools for PP (e.g. screening questions to ask; frequency of screening; follow-up actions; definitions of risk factors or cut-off values that dictate specific actions; etc) and other details are available upon request

*ASSIST* Alcohol, Smoking and Substance Involvement Screening Test, *BMI* Body Mass Index; BP: Blood pressure, *CVD* Cardiovascular disease, *eGFR* Estimated glomerular filtration rate, *ENDS* Electronic Nicotine Delivery Systems, *GAD-2* Generalized Anxiety Disorder 2-item, *HPV* Human Papillomavirus, *MOBP* Manual office blood pressure device, *PCP* Primary care provider, *PP* Prevention Practitioner, *STI* Sexually transmitted infection

The CDPS recommendations for heart disease and colorectal and breast cancers were only targeted to those deemed ‘high-risk’ (based on various clinical criteria such as family history) in the 18–39 age group. For most of the new topics, we also identified specific maneuvers or screening questions/tools that could be incorporated into the BETTER visits or into BETTER tools.

## Discussion

We used a structured search of published and grey literature, and a systematic search of specific databases to compile recent evidence from clinical practice guidelines on risk factors and individual prevention and screening actions relevant to adults aged 18–39 years, particularly those living with low income, in Canada. We also obtained input from our co-investigators, a team of experts in primary care, public health, the social determinants of health, and the BETTER program. Through this process, we were able to identify 42 recommendations within 21 total topics that will be applied in the BETTER LIFE approach for younger adults living with low income.

Some topics and health recommendations from previous BETTER versions were updated or included, such as those addressing *diabetes, cardiovascular disease, cancer, smoking, alcohol, nutrition, and exercise*. Risk assessments for *diabetes, cardiovascular disease* and most *cancers* were similar for those aged 18–39 years old as with previous versions of BETTER, though routine screening was only recommended for those deemed high risk (with the exception of cervical cancer screening). We found evidence-based guidelines addressing new topics relevant specifically to 18–39 year olds grouped into the following new domains: *healthy relationships and healthy sexuality, immunization, oral health, social determinants of health, and substance use*. Some recommendations in BETTER LIFE were similar to those published by others [97–99], though the recency, diversity, and sources of our search; our harmonization and implementation process, as well as the definition of our target population were different. For example, Persaud et. al. developed 15 preventive care recommendations and 1 policy recommendation that promote health equity in Canada. Although their work and ours both prioritize health equity in primary care, Persaud et al. did not have any age restrictions on their target population nor a primary focus on uptake of individual-level preventive actions. They also utilized systematic reviews, primary research articles and randomized controlled trials to develop recommendations using a GRADE approach. Because we prioritized recommendations that were individually actionable, supported by evidence that met our criteria, and ranked highly by content experts, topics like *vitamins* and *skin cancer* prevention were eventually omitted. Although we ultimately excluded *skin cancer*, this topic is an important one in many countries such as Australia [100].

Taking specific contexts into account is important when determining how best to implement and support uptake of the recommendations. For some new topics, we found stronger evidence for resources and screening tools for PPs than for specific recommendations (e.g. the National Institute on Drug Abuse (NIDA) Quick Screen or the Alcohol, Smoking and Substance Involvement Screening Test (ASSIST) for *substance use*). PPs identified local community resources for some new health topics (*parenting; substance use; oral health*) which could help to support participants achieve recommended actions. They also suggested considering social contexts as opportunities for engagement, e.g. by focusing conversations in BETTER LIFE visits on the concepts of health promotion or meaningful overall health and social well-being rather than explicit chronic disease prevention; by using different media for sharing health information (e.g. mobile apps, social media or online resources); by considering social contexts as barriers or enablers of behaviour change, especially regarding *physical activity, alcohol, substance use*; or by taking life stage into account (single adult vs. parenting).

Our study had several strengths and limitations. Our strengths include a rigorous critical appraisal of the literature with a two-step quality assessment process and independent review that ensured that only guidelines that met high methodological rigour and transparency were included for data extraction and harmonization; focus on actionable recommendations (e.g. goal-setting, access/referral to community resources); and meaningful collaborations with diverse community, public health, and clinical experts. However, all the guidelines were published prior to the COVID-19 pandemic, so did not take pandemic-related disruptions and health impact into account. COVID-19 has exacerbated health and economic inequities and disproportionately affected racialized and low income groups with a higher risk of exposure due to living and working conditions; higher prevalence of co-morbidities; inequitable access to testing and treatment; and disruption of health services [101, 102]. We also relied on consensus to resolve disagreements during the screening process and to formulate the final recommendations as well as on voluntary responses during harmonization which led to varied numbers of reviewers for each recommendation, and which may be subject to bias. However, we used AGREE-II to ensure transparency and careful documentation, and also consulted a wide and diverse range of experts (in primary care, public health, the social determinants of health, Prevention Practitioners, and the Community Advisory Committee) at many stages of the project. Finally, we may have missed guidelines because we targeted our search to specific criteria, repositories, and databases.

## Conclusion

Adults living with low income are at increased risk of chronic disease. Through critical literature review and guideline harmonization, we have curated a list of individual-level actionable recommendations relevant to prevention and screening for people aged 18–39 living with low income in English-speaking countries.

### Supplementary Information

Supplementary Material 1.

Supplementary Material 2.

Supplementary Material 3.

Supplementary Material 4.

## Data Availability

Data sharing is not applicable to this article as no datasets were generated or analysed during the current study.

## Abbreviations

ACE: Adverse Childhood Experiences
ADAPT-ITT: Assessment, Decision, Adaptation, Production, Topical Experts, Integration, Training, Testing
AGREE-II: Appraisal of Guidelines for Research & Evaluation II
ASSIST: Alcohol, Smoking and Substance Involvement Screening Test
AUDIT-C: Alcohol Use Disorders Identification Test
BETTER: Building on Existing Tools To Improve Chronic Disease Prevention and Screening in Primary Care
BETTER WISE: Building on Existing Tools to Improve Cancer and Chronic Disease Prevention and Screening in Primary Care for Wellness of Cancer Survivors and Patients
BMI: Body mass index
BP: Blood pressure
CAC: Community Advisory Committee
CDPS: Chronic disease prevention and screening
CINAHL: Cumulated Index to Nursing and Allied Health Literature
CTFPHC: Canadian Task Force on Preventive Health Care
COVID-19: Coronavirus disease 2019
CVD: Cardiovascular disease
eGFR: Estimated glomerular filtration rate
ENDS: Electronic nicotine delivery systems
GAD-2: Generalized Anxiety Disorder 2-item
HPV: Human papillomavirus
MOBP: Manual office blood pressure device
NIDA: National Institute on Drug Abuse
PHAC: Public Health Agency of Canada
PCP: Primary care provider
PP: Prevention Practitioner
RCT: Randomised control trial
STI: Sexually transmitted infection
US: United States
