# Supplementary figures and images for: BETTER LIFE- guidelines for chronic disease preventive care for people aged 18–39 years: a literature review
Source: BMC Prim Care. 2024 Jun 22;25:224. doi: 10.1186/s12875-024-02471-9 (PMC11193284; doi:10.1186/s12875-024-02471-9)

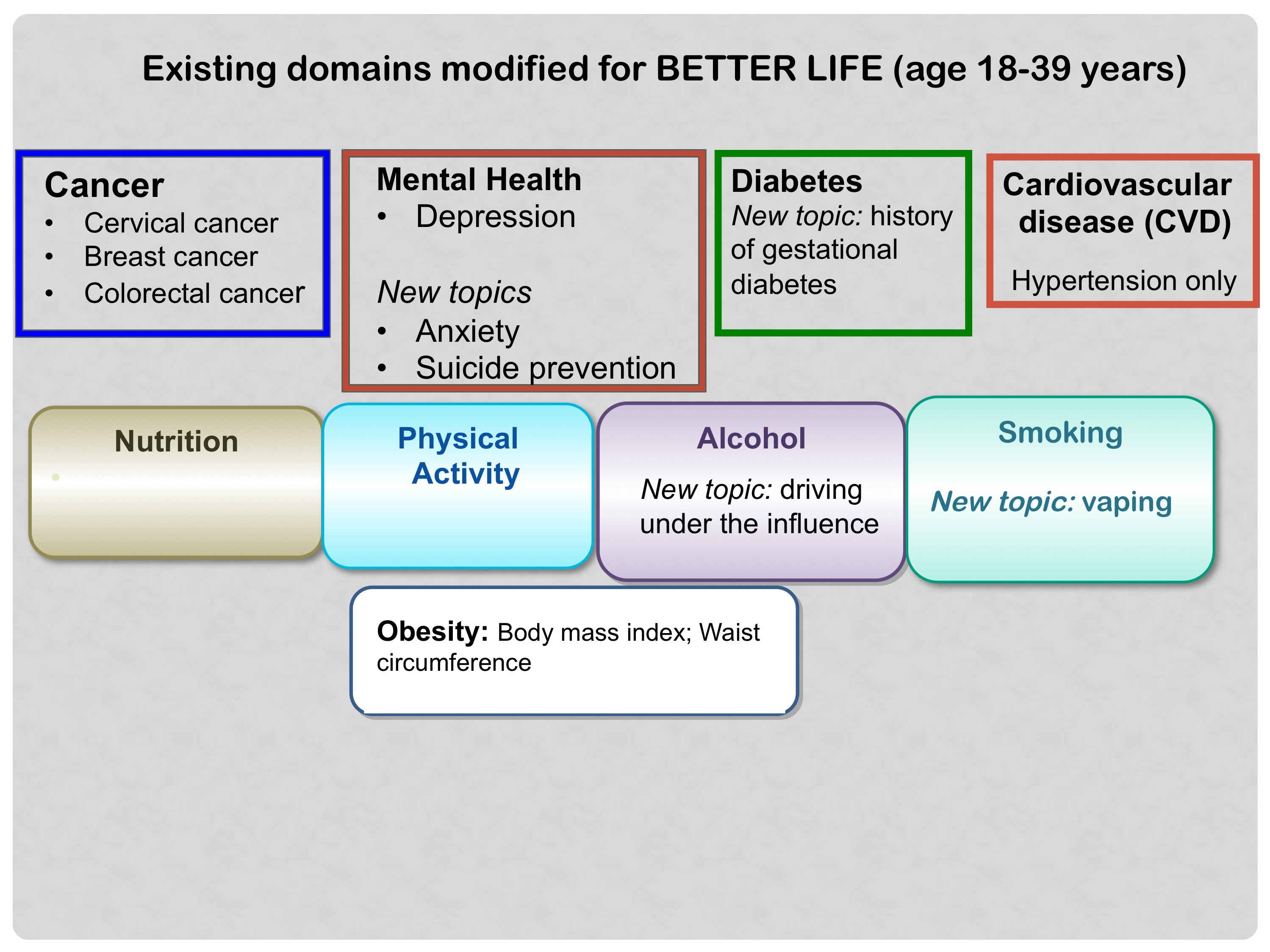

Supplement: Supplementary file 1 — Supplementary Material 1. [file 12875_2024_2471_MOESM1_ESM.jpg]

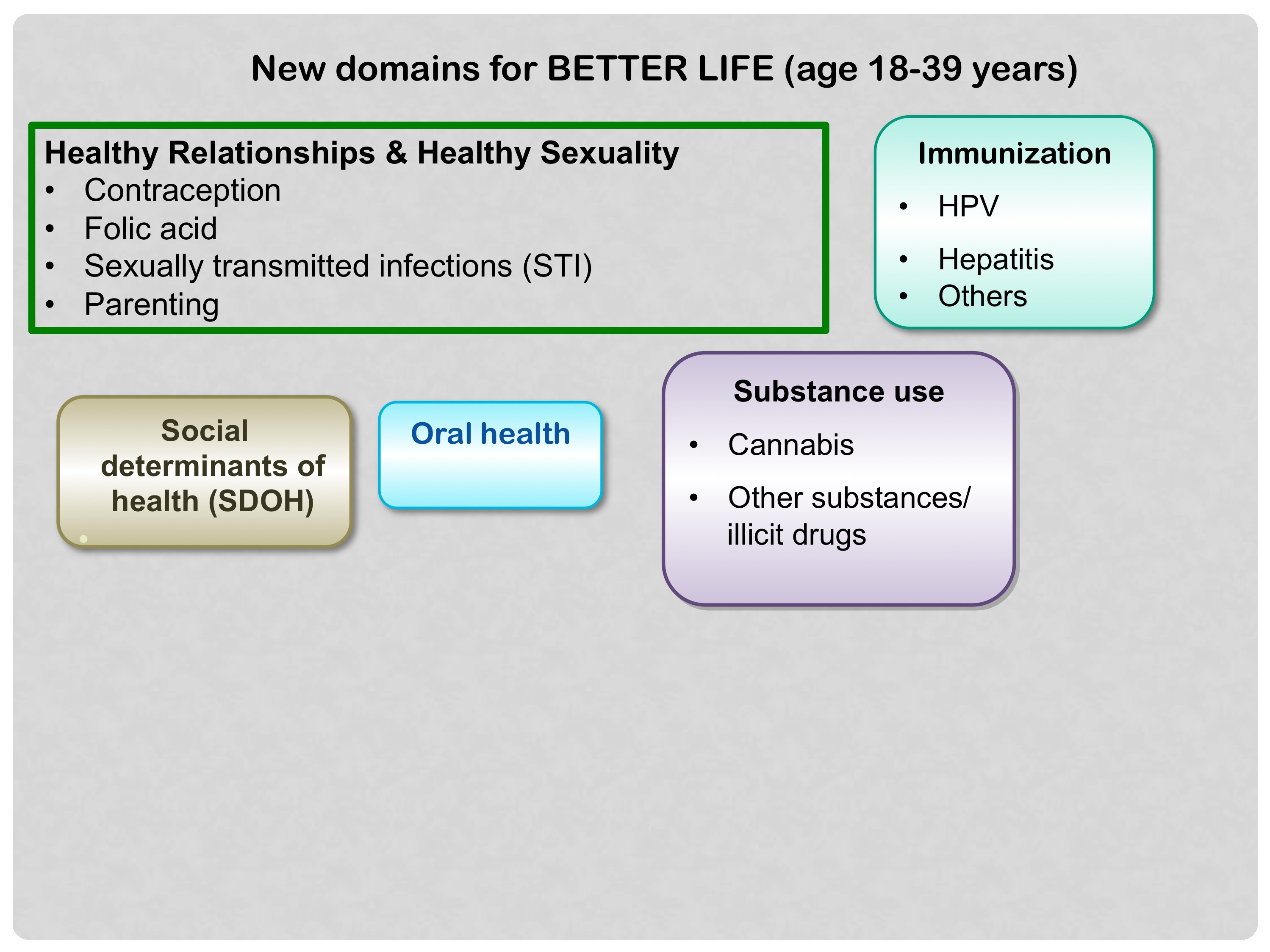

Supplement: Supplementary file 2 — Supplementary Material 2. [file 12875_2024_2471_MOESM2_ESM.jpg]
